# Toxicity and Repellency of (*E*/*Z*)-3-Butylidenephthalide: A Natural Compound Isolated from *Ligusticum porteri* Root Extract Evaluated Against Imported Fire Ants (Hymenoptera: Formicidae)

**DOI:** 10.3390/insects15110828

**Published:** 2024-10-23

**Authors:** Abbas Ali, Farhan Mahmood Shah, Ikhlas A. Khan

**Affiliations:** National Center for Natural Products Research, The University of Mississippi, Oxford, MS 38677, USA; fshah@olemiss.edu (F.M.S.); ikhan@olemiss.edu (I.A.K.)

**Keywords:** imported fire ants, toxicity, repellency, (*E*/*Z*)-3-butylidenephthalide, digging behavior

## Abstract

Imported fire ants are pests of significant importance, especially in the southern United States. We tested (*E*/*Z*)-3-butylidenephthalide, a natural compound that was isolated from the ethanolic extract of *Ligusticum porteri* roots, as a repellent and toxicant against workers of imported fire ants. A series of serial concentrations, starting from 156 µg/g until failure, were tested using digging bioassays. Workers removed significantly less sand from the vials treated with (*E*/*Z*)-3-butylidenephthalide as compared to the ethanol control. Based on sand removal data, (*E*/*Z*)-3-butylidenephthalide treatment resulted in a more significant digging suppression against red imported fire ant workers at concentrations ranging between 19.5 and 0.6 µg/g than the solvent control whereas sand removal at 0.3 µg/g was similar with the solvent control. Black imported fire ants showed repellency at serial concentrations ranging between 19.5 and 0.15 µg/g whereas the hybrid imported fire ants showed repellency between 19.5 and 4.9 µg/g. In DEET treatments, red and black imported fire ants showed repellency at dosages of 125 to 62.5 µg/g, whereas the treatment failed at the dose of 15.6 µg/g in hybrid fire ants. (*E*/*Z*)-3-butylidenephthalide with LC_50_ values of 11 and 16.4 µg/g was toxic against red and black imported fire ants, respectively, followed by hybrid imported fire ants (LC_50_ = 104.7 µg/g). Fipronil with LC_50_ values of 0.49, 0.33, and 0.53 µg/g against red, black, and hybrid fire ants, respectively, was more toxic than (*E*/*Z*)-3-butylidenephthalide. In residual bioassay, toxic activity lasted for 3 weeks at dosages of 250 and 500 μg/g against HIFA. The high repellency and toxicity of (*E*/*Z*)-3-butylidenephthalide against imported fire ants makes it a natural compound of interest for further evaluation under natural field conditions.

## Simple Summary

We tested (*E*/*Z*)-3-butylidenephthalide, a natural compound that was isolated from the ethanolic extract of *Ligusticum porteri* roots, for repellency and toxicity against workers of imported fire ants. In our digging bioassays, workers removed significantly less amount of sand from vials containing sand treated with (*E*/*Z*)-3-butylidenephthalide when compared to the ethanol control. Based on sand removal, red imported fire ant workers showed a digging suppression up to the lowest concentration of 0.6 µg/g in (*E*/*Z*)-3-butylidenephthalide as compared to the control solvent. Black imported fire ants showed repellency at the lowest concentration of 0.15 µg/g whereas hybrid imported fire ants showed repellency at the lowest concentration of 4.9 µg/g. In red and black imported fire ants, DEET was active up to the concentration of 62.5 µg/g, whereas the treatment failed at 15.6 µg/g in hybrid fire ants. (*E*/*Z*)-3-butylidenephthalide showed LC_50_ values of 11 and 16.4 µg/g against red and black imported fire ants, respectively, followed by an LC_50_ of 104.7 µg/g in hybrid fire ants. Fipronil with LC_50_ values of 0.49, 0.33, and 0.53 µg/g against red, black, and hybrid fire ants, respectively, proved to be more toxic. This study provides valuable biological information on this natural product against imported fire ants, emphasizing further testing under natural field conditions.

## 1. Introduction

Red imported fire ants (RIFAs), *Solenopsis invicta* Buren, black imported fire ants (BIFAs), *Solenopsis richteri* Forel, and their hybrids (HIFAs) are significant pests, especially in the southern United States. Both red and black fire ants were introduced from South America to the United States in the early 20th century [1]. Currently, red imported fire ants have been reported in 13 states and Puerto Rico [2], whereas black fire ants are found in smaller niche areas [1,3,4]. Hybrid imported fire ant (HIFA) population boundaries are widely distributed in Alabama, Arkansas, Georgia, Mississippi, and Tennessee [1,3,4,5,6,7]. Because of favorable temperatures, fire ant populations explode rapidly, especially during spring and fall, and rainy and humid temperatures during summer in the southern USA.

Synthetic pesticides are mostly used to manage imported fire populations [8], including the use of contact insecticides through direct treatment of the mounds as well as the baits. However, frequent mound relocations warrant multiple treatments [9]. The negative impacts resulting from repeated, long-term use of toxic synthetic pesticides have shifted the focus of researchers towards exploring relatively nontoxic natural products [10]. Secondary plant metabolites are extensively used in cosmetics, foods, and as pharmacological additives for flavors and fragrances [11]. Many naturally occurring compounds have been reported to be effective repellents and toxicants. Monoterpenoids are reported to be neurotoxic [12]. The use of repellents can be very useful against fire ants in sensitive areas like nursing homes, schools, and hospitals. Imported fire ants can effectively be prevented from re-entering treated materials in nursery stock and equipment by using effective repellents. Imported fire ants are reported to damage electrical equipment and circuitry while digging around these structures [13]. To stop the fire ants from spreading to non-infested areas, a federal quarantine law has been in force against imported fire ants in the USA (http://www.aphis.usda.gov/ppq/ispm/fireants/index.html, accessed on 29 August 2024).

Several natural products have been reported to suppress fire ant digging behavior [14,15,16,17,18,19,20]. Octanoic acid has been reported to be effective in removing fire ant infestation from treated nursery plant pots [16]. Sage (*Saliva* spp.) leaves, pine (*Pinus* spp.) needles, and water suspensions of cedar shavings were effective fire ant repellents [18]. Vogt et al. [21] reported that the toxicity of citrus oil formulations with d-limonene was similar to conventional insecticides, and Appel et al. [22] confirmed the repellency and toxicity of mint oil granules against RIFA workers in mounds under field conditions. Chen [19] reported the repellency of dimethyl and diethyl phthalates against RIFAs.

At NCNPR, we are running a screening program and continuously screening natural products for their toxicity and repellency against mosquitoes and imported fire ants. The ethanolic extract of *Ligusticum porteri* J.M.Coult. & Rose (Apiaceae) roots, a herbaceous flowering plant native to the Rocky Mountains, showed strong repellency against mosquitoes. Two isomers, (*Z*)-3-butylidenephthalide, (*E*)-3-butylidenephthalide, and a mixture (*E*/*Z*)-3-butylidenephthalide, were identified as the compounds responsible for the repellency of this extract against mosquitoes. (*E*/*Z*)-3-butylidenephthalide also showed increased repellency in combination with carotol against mosquitoes [23]. The repellency and toxicity of (*E*/*Z*)-3-butylidenephthalide against imported fire ants have not been documented. Based on mosquito repellency data against mosquitoes, we tested (*E*/*Z*)-3-butylidenephthalide to determine its toxicity and repellency against imported fire ants. This study reports the toxicity and repellency of (*E*/*Z*)-3-butylidenephthalide against imported fire ants.

## 2. Materials and Methods

### 2.1. Chemicals

**Figure 1:**
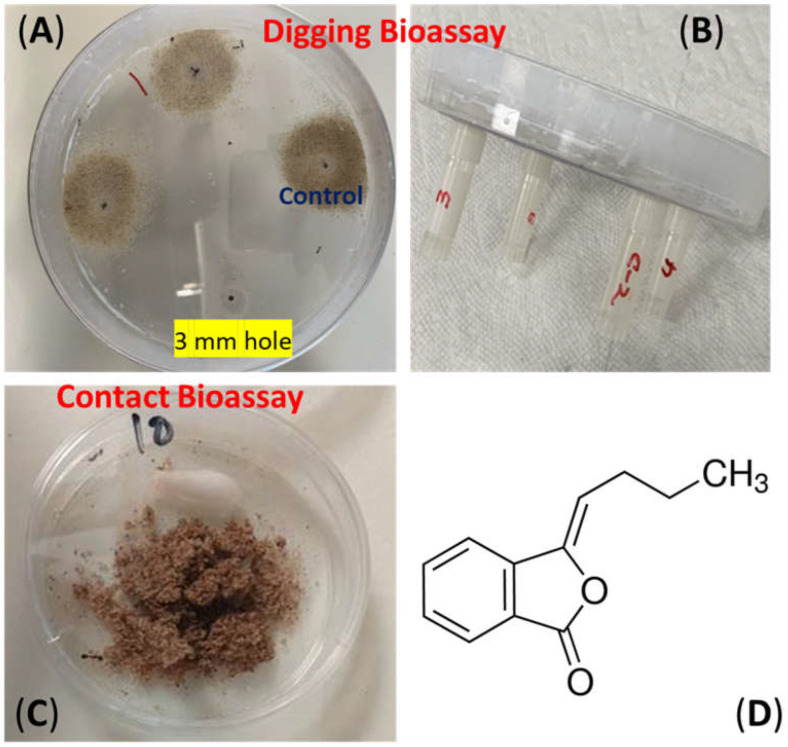
Imported fire ant bioassay setups: digging bioassay for repellency (**A**,**B**); contact bioassay for toxicity; (**C**) and chemical structures of (*E*/*Z*)-3-butylidenephthalide (**D**).

(*E*/*Z*)-3-butylidenephthalide CAS # 551-08-06 (Figure 1D) was purchased from SAFC, St. Louis, MO, USA 63178. DEET CAS # 134-62-3 and fipronil CAS #120068-37-3 were purchased from Sigma-Aldrich, 3050 Spruce Street, St. Louis, MO 63103, USA.

### 2.2. Fire Ants

(*E*/*Z*)-3-butylidenephthalide was screened and tested for its toxicity and repellency against imported fire ants using a set of bioassays. RIFA, BIFA, and HIFA workers were used in these bioassays. BIFA colonies were brought from Tunica County, MS-713, Hernando, MS 38632 (34°49′56.5″ N 90°12′55.6″ W). RIFAs were collected from Washington County, MS 38748 (33°09′31.2″ N 90°54′56.4″ W). HIFA workers were from natural mounds located at University Field Station (University of Mississippi, 15 County Road 2078, Abbeville, MS 38601, USA). Ants collected from different locations were kept in the original mound soil in talcum powder-lined plastic buckets to prevent their escape. Crickets and a 25% sugar–water solution were used as a source of food. A test tube filled with water and plugged with cotton supplied the moisture. The ants were maintained under laboratory conditions of 32 ± 2 °C temperature and 50% ± 10% relative humidity for one month before starting the bioassays. The ant species and hybrid identification was based on venom alkaloid and hydrocarbon indices of workers [24,25].

### 2.3. Digging Bioassay

Repellency against imported fire ants was assessed using a digging bioassay (Figure 1A,B). Fire ant workers start digging when released in a suitable digging substrate like moistened sand. The digging bioassay assumes that workers do not dig, or that digging is relatively low in repellent-treated sand versus untreated controls. Instead of a commonly generalized definition of the term repellent, more precise terminology is used to describe fire ant behaviors such as olfaction and contact responses. The repellency in fire ants can also be defined as the ability of the chemical/compound to suppress worker digging behavior, generally referred to as the suppression of digging ability in response to repellent treatments [19]. The repellency of (*E*/*Z*)-3-butylidenephthalide was determined using a digging bioassay described by Ali et al. [24]. Briefly, the repellency bioassay consisted of a 150 mm × 15 mm Petri dish arena and four 2 mL Nylgene Cryoware Cryogenic vials with caps (Figure 1B). The inner walls of the arena were made escape-proof after coating with Insect a Slip. A total of 4 grams of sand was weighed in a 45 mL fluted aluminum dish and treated in a volume of 400 µL. Both the stock solution and serial dilutions were prepared in ethanol. Controls were treated with ethanol alone. The solvent was evaporated, and the sand was moistened by adding de-ionized water at a rate of 0.65 µL/g of sand. The vials were then tightly filled with treated sand. Each vial contained a mean of 3.6 g of sand measured on a dry weight basis. The vials were screwed to their lids at the bottom of the arena and fifty workers were released in the center of the arena (Figure 1A). After 24 h, the vials were unscrewed, and left-over sand from these vials was collected back into aluminum dishes, dried at 150 °C for 1 h, and weighed. A series of concentrations were tested until the sand removed in a treatment became similar to the control solvent. Each experiment was replicated 3 times on 3 different days. The ants were not fed during these experiments. Data were analyzed using an Analysis of Variance and the mean, were separated using the Ryan–Einot–Gabriel–Welsch multiple range test at *p* ≤ 0.05 (SAS 9.4 (2012)).

### 2.4. Residual Digging Suppression

Bioassays were conducted to determine the dose-dependent residual digging suppression of (*E*/*Z*)-3-butylidenephthalide. Briefly, the bioassay consisted of two 100 g plastic cups. In total, 30 g of the mixture (75% 500-micron-sized sand and 25% mound soil) was weighed in 45 mL fluted aluminum weighing dishes, and treatment was applied in a volume of 3 mL (100 µL/g). After the evaporation of the solvent, the sand was moistened with water at a rate of 0.65 µL/g. The treated sand was then transferred into 100 g plastic cups. The control was treated with ethanol, the control solvent. These cups were placed in a tray (15 W × 23 L × 5 H cm) with borders lined with Insect a Slip. Fifty workers were released in each tray and their movement was observed. This bioassay works on the principle that fire ant workers move from dry to moist sand to avoid dehydration. Since initial moisture levels in the control and the treatment are the same, workers first move to the control where they dig tunnels and nest as per their behavior. On day 2, when the sand dried in these cups, only cups with the treatments had water applied at a rate of 0.65 µL/g. Since the sand in the control dried, to avoid dehydration, the workers left the control cup. They moved out of the tray. These workers were left with two choices: either to move into the treated cup that had moisture or stay in the tray to die of dehydration. A range of serial dosages from 500 to 62.5 µg/g were tested. The treatments were prepared as water emulsions. The ants were not fed during the experiments. In our preliminary assays, we noticed that the workers did not enter sand treated with (*E*/*Z*)-3-butylidenephthalide because of repellency, and if they entered, they would become disoriented and die because of the toxicity depending on the concentration of the treatment.

### 2.5. Toxicity Bioassay

The toxicity of (*E*/*Z*)-3-butylidenephthalide against imported fire ant workers was determined using a contact toxicity bioassay (Figure 1C) that uses sand as a digging substrate. Details of this bioassay are described by Ali et al. [24]. Various concentrations of (*E*/*Z*)-3-butylidenephthalide and fipronil, a positive control, were incorporated into a measured quantity of 500-micron-sized sand. Fipronil is the main ingredient of many commercially available baits used against imported fire ants. In this bioassay, fire ants dig through the sand as soon as they are exposed to it and their movement through the treated sand exposes them to natural products. Stock solutions and dilutions were prepared in ethanol. In total, 3 grams of sand was treated in a volume of 300 µL of ethanol in 42 mL fluted aluminum dishes. The sand in the control was treated with ethanol alone. After evaporating the solvent, the sand was moistened by using de-ionized water at a rate of 0.65 µL/g of sand. The treated sand was transferred into a 60 × 15 mm Petri dish with inner walls coated with Insect a Slip. Ten workers were released per treatment. The dish was covered with a lid to avoid drying the sand and a water-soaked cotton swab with the tip soaked was placed in the dishes to ensure a continuous supply of moisture. The numbers of dead ants were counted at 24 h post-treatment. Any ants that were severely moribund and incapable of moving when touched using a hairbrush were noted as dead. LC_50_ values were calculated using Probit analysis (SAS 9.4 (2012)) [26].

## 3. Results

### 3.1. Digging Bioassay

Table 1 presents the mean weight (g) of treated sand removed by workers of two species and hybrids in the digging bioassay with different concentrations of (*E*/*Z*)-3-butylidenephthalide and DEET. RIFA workers removed significantly less sand from (*E*/*Z*)-3-butylidenephthalide treatments at the concentrations of 19.5, 9.8, 4.9, 2.4, 1.2, and 0.6 µg/g than the solvent control, whereas the amount of sand removed at 0.3, 0.15 and 0.075 µg/g was similar to the solvent control. BIFA showed repellency at serial dosages ranging between 19.5 and 0.15 µg/g as compared to solvent control whereas the HIFA workers showed repellency at serial dosages ranging between 19.5 and 4.9 µg/g. DEET treatments showed repellency at dosages of 125 and 62.5 µg/g against red and black imported fire ants, whereas the treatment failed at 15.6 µg/g in hybrid imported fire ants.

### 3.2. Toxicity Bioassay

Based on mosquitoes’ data, this compound was screened for toxicity against imported fire ant workers which showed toxicity. (*E*/*Z*)-3-butylidenephthalide was further replicated to develop dose–response curves. Toxicity data are presented in Table 2. (*E*/*Z*)-3-butylidenephthalide with LC_50_ values of 11 and 16.4 µg/g was very toxic against red and black fire ants followed by hybrid imported fire ants (LC_50_ = 104.7 µg/g). Fipronil with LC_50_ values of 0.49, 0.33, and 0.53 µg/g in red, black, and hybrid fire ants, respectively, was more toxic than this compound.

### 3.3. Residual Digging Suppression

**Figure 2:**
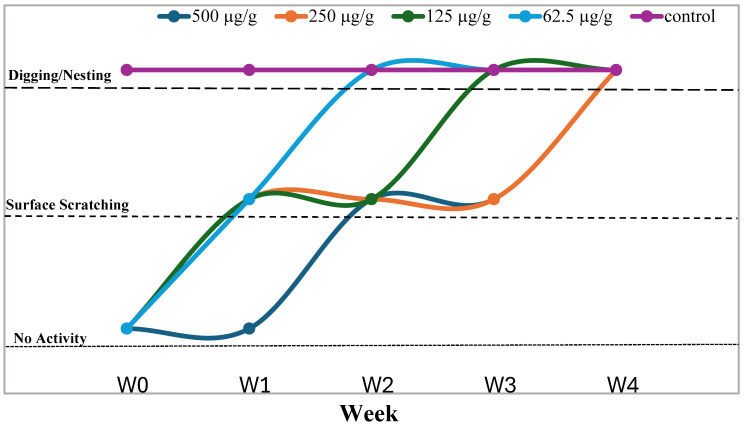
Dose (µg/g)-related residual repellency of (*E*/*Z*)-3-butylidenephthalide. No activity refers to the workers failing to dig or scratch the surface. Surface scratching indicated the state where the workers tried to scratch the surface but failed to dig. Digging/nesting refers to the stage where workers were able to successfully dig through and make the tunnels in the sand as they do normally in the untreated control. As the movement of workers was regulated by availability of the moisture, digging activity was a collective function of released workers, and every week, a new batch of workers was used, so it was not possible to quantify the activity.

Data on the residual digging suppression of (*E*/*Z*)-3-butylidenephthalide are given in Figure 2. (*E*/*Z*)-3-butylidenephthalide showed complete digging suppression up to 2 and 3 weeks from the time of application at concentrations of 62.5 and 125 µg/g, respectively. At dosages of 250 and 500 µg/g, digging suppression lasted up to 4 weeks.

## 4. Discussion

Many natural products have been reported as repellents against insects including mosquitoes and imported fire ants. Ethanolic extract of roots of *Ligusticum porteri* showed high repellency against mosquitoes [23]. Pure compounds (Z)-3-butylidenephthalide and (*E*)-3-butylidenephthalide, and a mixture, (*E*/*Z*)-3-butylidenephthalide, isolated from ethanolic extract of roots of *Ligusticum porteri* through bioassay-guided isolation showed repellency in in vitro and in vivo bioassays against *Aedes aegypti* [23]. Based on the repellency of (*E*/*Z*)-3-butylidenephthalide against mosquitoes, we tested this compound against imported fire ants for its biological activities.

The biological activity of repellents varies as a function of the concentration levels of treatment [26] in yellow fever mosquitoes, but in the case of imported fire ant workers, the repellency was not dose specific, and repellency was similar in different treatments until it reached the ranges of failure. Repellency and toxicity can play a complementary role in the management of imported fire ants. The repellency suppresses the digging ability of ants; the workers become confused and fail to carry on their normal digging behavior while toxicity kills these disoriented workers. The reduced removal of the treated sand in digging bioassays indicated that the treatment has suppressed the digging ability of the fire ants. Based on the amount of sand removed as a criterion of repellency, in this study, (*E*/*Z*)-3-butylidenephthalide showed significantly higher repellency than DEET. DEET failed at the doses of 62.5 to 15.6 µg/g in different species of fire ants whereas (*E*/*Z*)-3-butylidenephthalide showed repellency up to the dosages of 0.6, 0.15, and 4.98 µg/g in red, black, and hybrid imported fire ants, respectively, suggesting that repellency varied among fire ant species and hybrids as a function of dose. Ali et al. [23] reported the repellency of (Z)-3-butylidenephthalide, (*E*)-3-butylidenephthalide, and (*E*/*Z*)-3-butylidenephthalide against mosquitoes. These data against workers of imported fire ants corroborate the findings of Ali et al. [23] who reported the repellency of these compounds similar to DEET against mosquitoes. However, in imported fire ant workers of all the species tested, the repellency of this compound was much higher than DEET. In fire ants, in addition to repelling the ants, repellents negatively affect the digging behavior which prevents the ants from digging and establishing in treated soil, i.e., potting soil of the nursery plants. As such, the probability of ants being transported to new places with the nursery plants can be drastically reduced. Residual digging suppression is another attribute that can be explored to determine the use of this compound in the management of imported fire ants. Chen [19] reported the repellency of different phthalates in imported fire ants and reported the highest activity in methyl phthalate and diethyl phthalate. However, this is the first report on the repellency of (*E*/*Z*)-3-butylidenephthalide against imported fire ant species.

Data on the toxicity of (*E*/*Z*)-3-butylidenephthalide indicated high levels of toxicity as a natural product against imported fire ants. These data corroborate the findings of Ali (personal communications) who observed high mortality of (*E*/*Z*)-3-butylidenephthalide against mosquito larvae. Repellency and toxicity traits make natural products with dual action unique and effective tools for the management of imported fire ants. The repellency causes disorientation which blocks the workers from effectively digging through treated mounds while the toxicity kills them. The ability of (*E*/*Z*)-3-butylidenephthalide to negatively affect the digging behavior of workers makes it a suitable candidate for treating the potting soil mixtures to prevent the entry of fire ants in the potted soil used to transport nursery plant materials. It will prevent the spread of fire ants from one place to the other during the transportation of potted plants. Treating individual mounds by drenching or preventive spraying in the lawns may minimize the risk of fire ant infestation in the treated areas. The literature is scarce on the potential safety of this compound for use in human activity centers. Since (*E*/*Z*)-3-butylidenephthalide was isolated from *L. porteri* root extract which has been used traditionally to treat many diseases in human beings [27,28], the compound may be potentially safe to use around human activity centers.

## 5. Conclusions

(*E*/*Z*)-3-butylidenephthalide showed digging suppression and toxicity against fire ants. Digging suppression and toxicity properties of (*E*/*Z*)-3-butylidenephthalide make it a unique and effective tool for the management of fire ants. The behavioral changes caused by exposure to the repellents cause disorientation which suppresses the digging ability while the toxicity kills the ants. Further studies will be conducted to test the residual toxicity and digging suppression under field conditions to explore the potential of different (*E*/*Z*)-3-butylidenephthalide formulations as imported fire ant population management tools.

## Figures and Tables

**Table 1:** Mean weight (g) of treated sand removed by red, black, and hybrid imported fire ant workers released in multiple-choice digging bioassay with different concentrations of (*E*/*Z*)-3-butylidenephthalide and DEET.

Conc. (µg/g)	Mean ± SE ^†^	*F*-Value	*p*-Value	Mean ± SE ^†^	*F*-Value	*p*-Value	Mean ± SE ^†^	*F*-Value	*p*-Value
	RIFA			BIFA			HIFA		
(*E*/*Z*)-3-butylidenephthalide							-		
Control	0.91 ± 0.13 A	2.35	0.1488	2.41 ± 0.18 A	11.19	0.0031	-		
0.3	0.2 ±0. 11 A			0.35 ± 0.09 B			-		
0.15	0.64 ± 0.23 A			0.92 ±0.37 B			-		
0.075	0.83 ± 0.23 A			1.39 ± 0.28 AB			-		
Control	1.28 ± 0.03 A	29.54	0.0001	2.69 ± 0.18 A	26.62	0.0002	1.79 ± 0.09 A	2.62	0.1225
2.4	0.03 ± 0.03 B			0.73 ± 0.07 C			1.19 ± 0.11 A		
1.2	0.21 ± 0.10 B			1.19± 0.33 B			1.20 ± 0.32 A		
0.6	0.22 ± 0.06 B			1.67 ± 0.17 B			1.62 ± 0.14 A		
Control	1.3 ± 0.09			2.73 ± 0.007			2.02 ± 0.16 A	65.5	0.0001
19.5	0			0			0.21 ± 0. 08 B		
9.8	0			0			0.41 ± 0.11 B		
4.9	0			0			0.28 ± 0.02 B		
DEET									
Control	-			-			1.26 ± 0.19 A	0.24	0.87
15.6	-			-			0.98 ± 0.49 A		
7.8	-			-			1.37 ± 0.28 A		
3.9	-			-			1.16 ± 0.29 A		
Control	1.43 ± 0.19 A	16.24	0.001	1.38 ± 0.25 A	8.9	0.006	1.58 ± 0.11 A	9.71	0.005
125	0.08 ± 0.04 C			0.00 ± 0.00 B			0.42 ± 0.25 B		
62.5	0.74 ± 0.18 B			1.22 ± 0.04 A			0.87 ± 0.13 B		
31.25	1.14 ± 0.10 AB			0.79 ± 0.33 AB			0.84 ± 0.04 B		

**^†^** Sand removed is in grams. Each individual experiment consisted of three concentrations and a control. The means within a column in an experiment not followed by the same letter are significantly different (Ryan–Einot–Gabriel–Welsch multiple range test, *p* ≤ 0.05).

**Table 2:** Toxicity of (*E*/*Z*)-3-butylidenephthalide against workers of three species of imported fire ants at 24 h post-treatment.

Compound	*n*	Slope ± SE	LC_50_ (95% CI) *	LC_90_ (95% CI)	χ^2^	*df*
RIFA						
(*E*/*Z*)-3-butylidenephthalide	30	0.81 ± 0.10	11.0 (8.5–14.1)	50.9 (35.9–80.4)	63.3	22
fipronil	40	0.99 ± 0.27	0.49 (0.4–0.74)	1.78 (0.86–14.1)	13.6	19
BIFA						
(*E*/*Z*)-3-butylidenephthalide	30	0.84 ± 0.08	16.4 (11.9–23.1)	79.6 (50.7–160.0)	91.5	22
fipronil	40	1.02 ± 0.14	0.33 (0.28 -0.44)	01.12 (0.76 ± 2.12)	51.9	19
HIFA						
(*E*/*Z*)-3-butylidenephthalide	30	1.93 ± 0.28	104.7 (88.7–123.8)	203.6 (165.5–283.0)	45.9	13
fipronil	40	1.95 ± 0.44	0.53 (0.4 ± 0.74)	1.03 (0.75 ± 2.1)	19.35	22

* LC_50_ and LC_90_ values are in µg/g and 95% CI is the confidence interval.

## Data Availability

All data are contained within the article.
